# Molecular patterns and processes in evolving sociality: lessons from insects

**DOI:** 10.1098/rstb.2022.0076

**Published:** 2023-04-10

**Authors:** Seirian Sumner, Emeline Favreau, Katherine Geist, Amy L. Toth, Sandra M. Rehan

**Affiliations:** ^1^ Centre for Biodiversity and Environmental Research, Department of Genetics, Evolution and Environment, University College London, Gower Street, London WC1E 6BT, UK; ^2^ Department of Ecology, Evolution and Organismal Biology, and Department of Entomology, Iowa State University, Ames, IA 50011, USA; ^3^ Department of Biology, York University, Toronto, Canada M3J 1P3

**Keywords:** modes of evolution, sociality, collective behaviour, genomics, superorganismality, major transition

## Abstract

Social insects have provided some of the clearest insights into the origins and evolution of collective behaviour. Over 20 years ago, Maynard Smith and Szathmáry defined the most complex form of insect social behaviour—superorganismality—among the eight major transitions in evolution that explain the emergence of biological complexity. However, the mechanistic processes underlying the transition from solitary life to superorganismal living in insects remain rather elusive. An overlooked question is whether this major transition arose via incremental or step-wise modes of evolution. We suggest that examination of the molecular processes underpinning different levels of social complexity represented across the major transition from solitary to complex sociality can help address this question. We present a framework for using molecular data to assess to what extent the mechanistic processes that take place in the major transition to complex sociality and superorganismality involve nonlinear (implying step-wise evolution) or linear (implying incremental evolution) changes in the underlying molecular mechanisms. We assess the evidence for these two modes using data from social insects and discuss how this framework can be used to test the generality of molecular patterns and processes across other major transitions.

This article is part of a discussion meeting issue ‘Collective behaviour through time’.

## 1. Introduction

Social behaviour in nature takes many different forms, from murmurations of birds or flocks of sheep to self-organized foraging of ants or division of labour among the organelles of a cell. Such societies differ in the level of social complexity they exhibit, some are ephemeral and others permanent; but they are united by shared phenotypic traits, including cooperation, coordination of behaviour, communication and resolution of conflicts. Biologists have long pursued the quest to understand the mechanisms underpinning how such coordinated and cooperative behaviour arises within ecological timescales; examples of such progress are showcased in this special issue. By contrast, how the mechanisms regulating social behaviour have changed on evolutionary timescales is less well studied. Outstanding questions include: how are the mechanisms regulating social behaviour altered by selection in order that different forms of collective behaviour can evolve? In most social lineages, the most recent common ancestor was a solitary organism—e.g. a lone-nesting bird or mammal was the ancestral state of cooperatively breeding birds [1] and mammals [2]; solitary wasps were the ancestral states in the evolution of ants, bees and social wasps [3]. How are the genomes of these solitary ancestral states modified such that complex social behaviours can be effectively regulated? Is the coordinated behaviour of relatively loose, ephemeral social groups—like flocks of gregarious feeding birds—regulated by the same core molecular processes as the coordinated behaviour of obligatory social groups of ‘superorganisms'—like groups of foraging ants?

**Figure 1.**
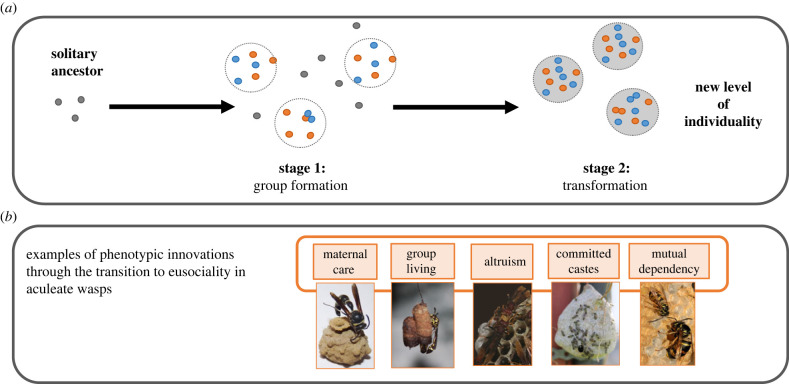
Stages in the major transition to insect superorganismality. (*a*) Formation of cooperative groups (stage 1) and the transformation of cooperative groups (stage 2) into a new ‘higher-level’ individual, composed of mutually dependent group members. Stage 1 species exhibit division of labour and cooperative brood care, but queens and workers differ only in behaviour not morphology, and workers retain reproductive totipotency. Castes in stage 2 species are determined during development such that queens are committed to reproduction and workers lack reproductive totipotency. (*b*) Examples of different phenotypic innovations that evolve during a major transition in wasps and bees. Innovations include the evolution of maternal care, where mothers care for brood (e.g. *Eumenes fraternus* (Eumeninae); photograph: Gary Budnik); group-living, where individuals live facultatively in groups (e.g. *Parischnogaster striatula* (Stenogastrinae); photograph: S. Sumner); altruism enables division of reproductive labour to be maintained among equally reproductively potent group members (e.g. *Polistes canadensis* (Polistinae); photograph: S. Sumner)*;* developmentally determined castes commit individuals to different roles (e.g. *Polybia occidentalis* (Polistinae); photograph: S. Sumner); specialized phenotypic states are mutually dependent on each other in the new level of individuality—the colony (e.g. *Vespula germanica* (Vespinae); photograph: C. Oi).

The evolution of complex sociality is one of the major transitions [4] whereby previously independent units unite to become mutually dependent components of a new level of individuality that represents a higher level of biological complexity—as a committed society [5]. Examples of major transitions include the evolution of eukaryotes from prokaryotes, multicellularity from single-celled organisms, and animal societies from solitary-living individuals; the latter is epitomized by the most complex societies of insects (ants, termites, some bees and wasps), whose colonies are sometimes referred to as ‘superorganisms' [4] (figure 1; Glossary). The transitions are diverse, describing the changing processes in genes, cells, behavioural strategies and physiology. For example, among animal societies the level of complexity in a society varies greatly, with some societies being simple, facultative collectives while others are partially or completely committed (irreversibly) to group-living. This diversity in social complexity may offer insights into the processes that shape the evolution of a social lineage and the major transition (figure 1). Despite this diversity, there are common traits across the transitions, associated with the concept that each transition involves the generation of complexity in the form of a society (e.g. organelles in a cell; cells in a body; insects in a colony). These include the emergence of irreversibly committed phenotypes within the group (e.g. queens and workers in insect colonies; tissue types in multicellular organisms), mutual commitment among the members within a group (e.g. into specialized reproductive and non-reproductive individuals/cells/tissues), and the generation of a new level of phenotype on which selection can act (e.g. worker sterility in social insects). These common analogies are compelling but remain largely conceptual. We lack an understanding of the evolutionary processes by which the major transition to sociality arises and specifically how these processes differ across the levels of social complexity [6]. Understanding these patterns across the putative evolutionary pathway(s) to sociality is important. This is because the assumptions we make about how complex life evolved will differ depending on the nature of these evolutionary processes [7].

Ever since Maynard Smith and Szathmáry's landmark publications on major transitions [4,8], biologists and philosophers of biology have sought to understand the mechanisms underpinning specific major transitions [9,10]. Debates have raged over the importance of mutations in protein-coding genes versus changes in gene regulation in the emergence of novel traits in the hierarchy of biological complexity [11,12]. A key reason for the disputes is that we lack a fine-scale dissection of the molecular processes that take place *during* the many stages of a major transition. In the absence of these data, the current literature assumes that changes associated with a major transition arise incrementally over time, via linear changes in the molecular processes that underpin the different stages in the evolutionary route to sociality [13,14]. An alternative, almost completely overlooked (or dismissed) hypothesis is that the evolution of sociality might involve large-scale mechanistic changes at the molecular level, resulting in nonlinear changes in the molecular processes that underpin the different levels of social complexity [15]. Such profound changes might be expected to coincide with the emergence of key social innovations, such as superorganismality itself.

We explore these ideas by suggesting a conceptual model on how evolution at the proximate level may give rise to various levels of social complexity at the ultimate level. Our thesis builds on an existing bedrock of literature that describes how phenotypes and levels of selection change during social evolution. We offer a predictive framework which poses tractable hypotheses on the molecular processes that underpin this major transition. Determining these proximate processes and the extent to which the processes are shared across the multiple independent origins of sociality may help us better understand the evolution of biological complexity more broadly. To explain our ideas and how they can be tested, we draw heavily on the extensive literature from the social Hymenoptera (ants, some bees and wasps) (figure 1). These social insects are excellent models for this because they include multiple independent origins of sociality [16,17] and they exhibit a range of social complexities, from the simplest forms of collective behaviour (where individuals make facultative decisions on whether and when to be social) through to superorganismality (societies where individuals are obliged to live in societies, where they are developmentally committed to a specific role—caste—during development, and where group members are mutually dependent on each other for reproduction).

## 2. Stages in the evolution of sociality

The major transition to superorganismality is characterized by a shift in the kinds of phenotypes that natural selection can act upon, generating new hierarchical levels that are often accompanied by a change in how information is transferred (or inherited) across generations. The products of the major transition—superorganisms—are some of evolution's most impressive phenomena, and worth studying in their own right to understand the complexity of life. However, in order to understand *how* the major transition arises, the putative stages of the transition must be defined and studied.

What are the ‘stages’ of the major transition to sociality? Using social insects as inspiration, Bourke [13] defined three stages in the evolutionary pathway of a major transition, specifically the transition from solitary living to superorganismality: group formation, group maintenance, and transformation into an obligate society composed of previously independent (related) individuals that are now incapable of living solitarily [13]. West *et al.*'s two-stage pathway can also be applicable to social insects [14], describing the formation of cooperative family groups (stage 1) and the transformation of such groups into a new ‘higher-level' individual, composed of mutually dependent group members, who are usually related to each other (stage 2) (figure 1*a*). Stage 1 is thought to represent a phase that the ancestors of ‘transitioned' species (i.e. those that have undergone the major transition) must have experienced. Accordingly, extant taxa that exhibit the characteristics of stage 1 may provide insights into the early stages of a major transition, when placed within an appropriate phylogenetic context [13,18–23]. Importantly, stage 1 species (e.g. family groups of insects) are regarded as evolutionarily stable states in their own right [24]—they are not assumed to be on an inevitable trajectory towards stage 2; moreover, such simple forms of sociality may not be required in order to evolve superorganismality [17,25,26] and it is possible that some stage 1 species may in fact be limited in their evolutionary potential by some molecular processes. Nonetheless, many stage 1 type species have been widely considered as useful models for inferring the conditions under which group formation could have evolved and is maintained [18–21].

Stage 2 biological units are ‘transitioned' to the new level of complexity: this has been described aspast the ‘point of no return' [17,25], with the commitment to irreversibility being the key defining feature of a major transition [13] (figure 1*a*). Stage 2 species are of interest in understanding evolutionary processes at the new level of individuality. Accordingly, comparing a range of phenotypes displayed by social insect species of stage 1 and stage 2, within a phylogenetically controlled framework, offers the opportunity to dissect the nature of the evolutionary process. Phenotypic traits considered in a phylogenetic context can help infer the evolutionary processes that a given lineage may have experienced and the key phenotypic traits that may characterize the major transition.

The distinction of two [14] or three [13] or even 12 [27] stages in a major transition is not intended to infer a di/tri/multi-chotomy in the process, but it is helpful in capturing the main evolutionary changes that are likely to occur in a major transition. These include the shift in levels of selection (i.e. from single- to multi-level selection of biological units), the shift in the balance of reproductive conflicts (i.e. from high conflict among competing (though related) ‘cooperators', to low conflict among mutualistic dependents with aligned fitness interests) and the emergence of key phenotypic traits (such as communication, specialization and recognition) required for maintenance of the higher society. In the major transition to superorganismality in insects, the shift from solitary living with ancestral maternal care traits to cooperative family groups with altruism represents stage 1 [28]; and the shift to committed, irreversible roles, division of labour with mutual dependency among members and the ability to coordinate cooperation at the level of the colony [29] (i.e. superorganism) can be considered as stage 2 (figure 1*b*, for wasps as an example).

## 3. Evolutionary arguments for linear and nonlinear molecular mechanisms in the evolution of sociality

### (a) Incremental changes in the evolution of social complexity imply linear changes in the molecular mechanisms regulating social behaviour

The prevailing hypothesis provided by evolutionary theory (formulated first by Darwin [30]) is that many small-scale micro-evolutionary molecular processes give rise to profound macro-evolutionary patterns; this hypothesis assumes a gradual process of molecular evolution without step-changes [31–33]. By extension, it is assumed implicitly that changes associated with a major transition, such as the evolution of sociality, arise incrementally over time, via a gradual evolutionary route [14,17] (as implicated in behavioural ecology's `phenotypic gambit' [7]); such a gradual process should be detectable as small ‘step-wise' linear changes in the genome-to-phenotype relationship, which arise gradually over evolutionary time via many small-scale changes in the genome (e.g. point mutations, gene expression, gene regulation) [34]. Incremental changes in the molecular mechanisms underpinning phenotypes will be advantageous if there are selective benefits to retaining phenotypic flexibility and reversibility [35]. A linear mechanistic route to a major transition appears evident at the phenotypic level: e.g. the ‘sociality spectrum’ observed in social bees and wasps, where there are extant species representing seemingly small differences in the complexities of their societies [18,20] (figure 1*b*). This route implies a gradual change in the target of selection, from the lower-level individual (e.g. the solitary insect) to the new higher-level ‘individual' (e.g. the group/society). For species representing intermediate forms of social complexity, selection would act at both levels of individuality (e.g. the individual insect and the individual colony) [36]. The implicit assumption that the evolution of sociality occurs incrementally via linear changes in the proximate mechanisms that regulate the behaviours of phenotypes in social groups is not surprising, as it is the most parsimonious explanation.

### (b) Step-changes in the evolution of social complexity imply nonlinear changes in the molecular mechanisms regulating social behaviour

An alternative hypothesis is that the evolution of new levels of social complexity requires step-changes in the molecular processes regulating social behaviour. Step-changes in evolution (sometimes described as punctuated evolution) are typically used to describe changes at the level of the phenotype, e.g. species diversification events like the Cambrian Explosion and early insect diversification [37–39]. Importantly, these punctuated evolutionary events are not necessarily characterized by one single ‘catastrophe' event; rather, they can be a series of profound events that may also be accompanied by periods of gradual change [37,40].

The ‘point of no return’ (stage 2 in the major transition), with an irreversible, new level of individuality (e.g. the eusocial colony), may be interpreted as a step-change evolutionary event at both the level of the phenotype and the level of selection [15]. It is entirely reasonable, therefore, to posit that this fundamental shift in phenotype and level of selection requires step-wise changes in the molecular mechanisms that regulate the social behaviours involved, resulting in nonlinear patterns in the molecular regulation of social behaviours at different levels of social complexity across the major transition. There will be selection for such nonlinear patterns if inflexibility and irreversibility are advantageous in the functioning of higher levels of individuality. This process might be exactly what is needed to enable the change in levels of selection that characterize the major transition to superorganismality [41,42]. As an example, Boomsma & Gawne [17] proposed that, although many species display the hallmarks of ‘classic' insect sociality, they do not express the specific set of traits that indicate a major transition (i.e. mutual dependency; committed (irreversible) castes) [17]. At the ultimate level, the major transition to superorganismality can only evolve when the fitness landscape is right: a family group headed by a lifetime-committed monogamous mother provides the inclusive fitness payoffs required for the evolution of committed altruists [25]. Commitment to such lifetime monogamy may require a profound change in the molecular mechanisms regulating mating and breeding behaviour. The requirement for a profound shift in molecular machinery to generate the right fitness conditions for the caste commitment to evolve would help explain why superorganismality is relatively rare, clade-specific and always phylogenetically irreversible; for example, pre-imaginal caste determination may have appeared suddenly (and irreversibly) in only one lineage of vespid wasps [26,43]. These studies raise the intriguing question of whether the mechanisms regulating different levels of social complexity across the major transition to superorganismality include nonlinear differences in underlying mechanisms rather than the gradual tweaking of subtly different ancestral ground-plans over many, small step-wise changes.

The step-wise route to sociality makes several important assumptions that are not supported by an incremental route. Firstly, the genomic processes of selective importance in the transition will only be apparent in species that are on the cusp of the transition itself; this means that processes regulating the simplest forms of sociality (i.e. in stage 1) and the forms of social complexity in species that have evolved from a superorganismal ancestor (i.e. beyond the ‘point of no return' (i.e. stage 2)) may be largely evolutionarily neutral, *with respect to the major transition*. Secondly, the molecular processes underpinning the relevant traits (e.g. cooperation; division of labour; communication; monogamy) in simpler societies (i.e. those which have evolved from ancestors that have not passed the ‘point of no return') will be different from the processes underpinning equivalent traits in the irreversibly committed superorganismal societies (i.e. those that have evolved from ancestors which have passed the ‘point of no return’). If this is true, then superorganismal species can tell us little about the *process* of the major transition, only about what happens in species that have evolved from a superorganismal ancestor. What happens afterwards allows us to understand how selection acts on traits at a new level of individuality (i.e. the society rather than the individual), but it tells us little about the major transition *per se* [25]. The implications of this insight on how comparative research should be conducted is more profound than is currently recognized, especially for the large body of research on the major transition in insects, where most of the key model organisms for sociogenomic studies (e.g. honeybees; ants [44]) are superorganisms: these may not be useful for understanding how (and why) major transitions arise [18,20,22,45,46].

## 4. Evidence from insects for linear and nonlinear patterns in the molecular processes regulating social behaviour across the spectrum of social complexity

The two fundamentally different (potentially co-occurring) evolutionary routes can be distinguished by dissecting the molecular processes that occur during a major transition [21,47]. In this section, we first draw up a list of some of the key molecular processes that could be considered signatures of linear or nonlinear events. Then, we survey the current literature on social insects in light of these two hypotheses.

**Figure 2.**
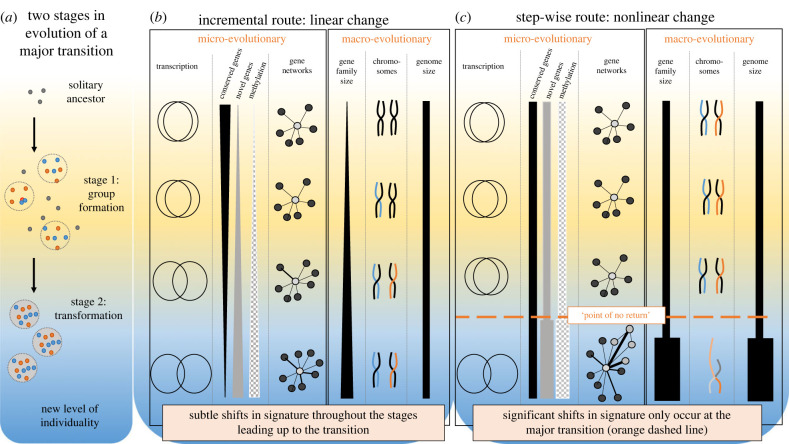
Examples of genomic signatures that would indicate a linear or nonlinear route to sociality. (*a*) Formation of cooperative groups (stage 1) and the transformation into a new ‘higher-level' individual, marked by irreversible commitment ('point of no return') to group-living (stage 2). (*b*,*c*) Two possible (non-mutually exclusive) routes across major transitions, a linear route (*b*, incremental changes in molecular processes) and a nonlinear route (*c*, step-wise shifts in molecular processes). Examples of small- and large-scale molecular signatures that may describe different stages of the transition are given. Small-scale changes include differential transcription (e.g. non-overlapping regions of the circles represent differentially expressed genes in different specialist phenotypes in the group (e.g. reproductive and non-reproductive entities in a social insect colony or a multicellular body)), the importance of conserved genes, novel genes and epigenetic processes in regulating the phenotypic innovations that occur through the transition, and gene networks of co-expressed/interacting genes. Large-scale processes include gene family expansions, chromosome rearrangements, synteny and genome size. Note these are examples only, and not intended to be an exhaustive description of the possible ways by which genomes may change during major transitions.

We loosely group the different types of genomic changes that can occur into two broad categories: small-scale (micro) genomic changes (small changes in transcription, gene regulation and gene interaction networks; point mutation; protein evolution) and large-scale (macro) genomic changes (gene family evolution; polyploidization events; genome rearrangements; large-scale deletions or insertions; genome duplications). Under this schema, small-scale changes are assumed to be *more often* (but not always) associated with incremental and small modulatory changes in phenotype, whereas large-scale changes are more often (but not exclusively) associated with step-change phenotypic shifts; empirical evidence supports this assumption [48,49]. These signatures provide a predictive framework for the molecular signatures of the two types of evolutionary processes (figure 2). Small- and large-scale genomic patterns can be detected by examining the molecular processes associated with phenotypic traits of individuals sampled from species representing different levels of social complexity, analysed within a phylogenetic framework; for example, between species that represent stages 1 and 2 of the major transition [18].

To determine the relative importance of linear and nonlinear molecular processes in the evolution of sociality, we require tractable models with extant examples representing the putative levels of social complexity that may characterize the transition (i.e. include stages 1 and 2), with multiple evolutionary origins to allow within- and between-phylogenetic comparisons, and for which multi-level genomic analyses (i.e. genome, tissue/life-stage/phenotype-specific transcriptomes, methylomes etc.) are feasible. The hymenopteran insects are rare in providing extant representatives of the different levels of social complexity, including solitary-living species which represent the ancestral state of the major transition, to species that live in family groups but retain autonomy, to species representing true superorganismality with irreversibly determined social roles. The individuals that make up societies show phenotypic specialization in the form of a division of reproductive labour: queen (reproductive) and worker (non-reproductive) castes are easily identifiable through measurable traits (e.g. egg-laying; maternal care); the phenotypic traits that indicate level of social complexity are well defined in terms of plasticity in ‘totipotency' (ability to adopt any role in the society), ‘commitment' (or irreversibility—inability to switch from one role (e.g. queen) to another (e.g. worker)) and ‘mutual dependency' (degree to which the individuals can function without each other). Moreover, the last 10 years has seen an explosion in the generation of multi-faceted genomic datasets (e.g. genomes, transcriptomes, epigenomes, proteomes) for a diverse and large array of social insect species [44], providing unprecedented opportunities to test predictions for both gradual and punctuated evolutionary routes using an empirical approach. Combined with the ease of conducting manipulative experiments on individual phenotypes for many of these species, these organisms present the required empirical tractability to test the predictions we propose (figure 2). The breadth and insights into the molecular basis of sociality provided by these studies have been recently reviewed, comprehensively, elsewhere [19]; instead, here we focus on the aspects of the current literature that are specifically relevant to determining the nature of the major transition, and what the next steps should be in providing the missing links in the process of the major transition in insect sociality.

### (a) Incremental evolution: evidence of linear changes in molecular processes

Under an incremental model of social evolution, new levels of complexity would be achieved by co-opting existing genomic machinery to produce novel social traits, possibly via a rewiring of regulatory processes (e.g. *cis*-regulation) that are shared across lineages: this is the evo-devo genetic toolkit hypothesis [11,50–52]. Any changes at the genomic level are expected to follow a linear trajectory in the different levels of social complexity across the major transition (figure 2*a*). Under this model, small-scale genomic changes (e.g. single nucleotide changes; subtle alterations in differential gene expression, gene networks and gene regulation) are likely to be common and correlate with the evolution of incremental and small modulatory changes in phenotype. These might manifest as linear patterns of increasing levels of differential gene expression between social phenotypes, along a gradient of social complexity. Other small-scale genomic changes include gradual rewiring of gene co-expression networks and cumulative changes in regulatory machinery. Large-scale genomic changes may also be detected under an incremental evolution model; these include incremental expansions of gene families, polyploidization events, genome rearrangements, large-scale deletions or insertions; they also include changes to chromosomal structure (e.g. chromosomal synteny, gene order and/or chromosomal insertions/deletions). Such changes are expected to accrue incrementally and at low, even, rates over long spans of evolutionary time. Thus, under a gradual mode of evolution, the molecular processes associated with the transition to sociality are predicted to follow a linear, incremental relationship (with gradual accumulation of small-scale and (more rarely) large-scale genomic shifts) across related taxa that represent different stages of the transition (figure 2*b*).

A prevailing hypothesis for the evolution of insect sociality is that there has been incremental decoupling (or differentiation of molecular processes) among the specialist phenotypes—queens and workers—across the major transition [18,23,33]. The decoupling hypothesis implies that the molecular mechanisms regulating social phenotypes in the simplest (stage 1) cooperative groups are inherited from the solitary ancestral state, and that these same phenotype-linked processes are ‘co-opted' and perhaps amplified in the more complex forms of sociality (stage 2) [51,53]. Additional modifications or elaborations are expected only in complex societies that have evolved from a superorganismal ancestor [19], but the incremental route predicts that the ‘point of no return' (at stage 2) requires no step-change in molecular machinery. There is overwhelming evidence for incremental changes in shared molecular processes across social insect species representing different levels of social complexity [18,19], indicating that linear changes in molecular processes across the major transition are likely to be important. Specific predictions can be derived from this rich genomic literature across organisms that implicitly assume an incremental route to evolving social complexity. This provides us with a bedrock of empirical and conceptual predictions of likely molecular signatures of an incremental route for the major transition [19,21].

In support of the incremental route, several studies have documented larger differences in gene expression between queen and worker castes between stage 2 (superorganisms) and stage 1 (simple societies) [47,54–56]. Subtle changes in the regulation of conserved genes are proposed to be major players, as many of the differentially expressed genes are associated with deeply conserved evolutionary processes such as energy metabolism, development, and transcriptional regulation [47,57–59]. For example, social insects representing stage 1 in the transition (e.g. *Ceratina* carpenter bees [56,60]; *Polistes* paper wasps [47,54,62,59,61]) exhibit very little brain transcriptional differentiation among castes, while comparable data on those representing stage 2 (e.g. *Apis mellifera* [63]*; Acromyrmex echinatior; Linepithema humile* [64]) differ by an order of magnitude more than those in stage 1. However, notably we lack such studies on species representing transitional stages between stages 1 and 2. Studies across multiple extant insect species exhibiting different levels of sociality suggest gradual rewiring of gene co-expression networks and cumulative changes in regulatory machinery [56,65,66] (figure 2*b*). Finally, the prominence of linear changes is consistent with the prevailing and overwhelming evidence of conserved sets of genes that appear to regulate social traits and caste across levels of sociality, and even across lineages—a ‘genetic toolkit' for sociality [21,56,58,64]. Together, these studies suggest some evidence for linear changes in genomic mechanisms underpinning the different stages of the major transition to superorganismality, involving incremental changes in gene regulation, co-option of deeply conserved genes, and expansions of ancient gene families across different levels of social complexity (figure 2*b*).

### (b) Step-wise evolution: evidence of nonlinear changes in molecular processes

Recent literature suggests that the scale of the mutation (e.g. a ‘small' single point mutation versus a ‘large' change such as an inversion of a chromosomal segment) is in fact linked to the scale of the expected phenotypic effect [48,49]. That is, large-scale genetic changes (e.g. those that involve whole genome duplication, gene duplication, changes in chromosomal arrangement, deletion or insertion of large stretches of DNA [67], or transposable element insertion into gene regulatory networks [68]) are more likely to have large-scale (often detrimental) consequences on organismal function. Such nonlinear changes in the molecular processes associated with sociality have recently been identified (e.g. supergenes; topologically associated domains), and several studies suggest that such large-scale genomic changes can result in major phenotypic changes that can sometimes be highly adaptive. A growing literature suggests large evolutionary innovations and transitions (e.g. radiation of angiosperms, diversification of animal body plans, plant domestication) may have been the result of major changes in genomes [67–74].

If different levels of social complexity evolve in a step-wise fashion across the major transition, then we expect this to be apparent in the form of nonlinear relationships between the rates of change in one or more molecular signatures with entities representing the different stages of the transition. We postulate that if any step-wise changes in molecular signatures occur, they are likely to coincide with the ‘point of no return' in the transition to stage 2, where levels of selection and levels of plasticity shift from the individual to include group-level effects and where selection of monogamous family groups is thought to be essential for achieving the transition to superorganismality (figure 2*c*). Such profound shifts do not necessarily have a signal that is measurable at the phenotypic level; analysis at the molecular level is likely to reveal insights that are not apparent from the phenotype. Large-scale genomic processes that may be putative signatures of punctuated molecular processes include significant genome rearrangement, genome duplication, large indels and chromoplexy (a combination of DNA translocation and deletions). Large-scale signatures of punctuated gene evolution include the appearance of orphan genes [75] with novelties in protein sequence, not just duplications or re-shuffled functional domains*.* Other signatures of step-wise evolution may include major shifts in transcriptomic gene regulatory networks, rapid evolutionary change and/or species diversification [76–78]. This literature has pinpointed genomic mechanisms such as bursts of transposable element insertion and concomitant epigenetic change, massive chromosomal rearrangements, drastic changes in gene copy number, and polyploidization events.

To date, the evidence for nonlinear changes in the molecular processes regulating different levels of social complexity in social insects is sparse; however, there is a general lack of sampling for the representative levels of social complexity in which one might expect to *detect* such step-wise changes in genomic machinery [15]. One exception is a recent study that used machine learning analyses of brain gene expression to classify castes from different species of social wasps that represent a broad spectrum of social complexity: suites of genes that correctly classified castes in species with simpler societies did less well at classifying castes in more complex societies, and *vice* *versa*, suggesting there may be different ‘genetic toolkits’ in play at different stages of social evolution [79]. The tendency for many (early) studies to focus on a gene-specific effect (e.g. [80]) may overlook the potential for more profound events such as evolution of ‘super-genes' [70]. A recent example of this is the gene *Gp-9*—an odorant-binding protein—which was thought to be the ‘master regulator' of social structure in the fire ant, *Solenopsis invicta.* More recent genome-wide analyses revealed that *Gp-9* is one of 616 genes on an entire ‘social chromosome', which is immune to recombination owing to a large inversion of 9 Mb [69]. Other studies have suggested key superorganismal traits are more likely to be associated with the emergence of novel genes [81,82], suggesting a possible role for new gene birth and possibly step-wise evolution in this major transition. In terms of large-scale genomic change, shifts from more conserved patterns of gene linkage and order (synteny), and profound innovations in the rearrangements of protein domains for transcription factor families, are predicted to accompany the transition to sociality, with evolutionary ‘ratchets’ limiting the potential for reversibility [83]. For example, ‘dramatic' changes in chromosomal structure and gene evolution (e.g. chromosomal synteny, gene order and/or chromosomal insertions/deletions) appear to characterize the evolution of the most complex of superorganismal insects [84,85]. Recent work has also suggested step-wise molecular evolution across two independent origins of sociality in corbiculate and carpenter bees [86]. The data suggest transitions to sociality are coupled with a dramatic rise in genome complexity, novel genes and protein evolution, as predicted by a step-wise route for a major transition.

## 5. Outstanding questions

Evidence for both linear and nonlinear molecular processes across the different levels of social complexity in the evolution of sociality makes sense since these processes are not expected to be mutually exclusive. However, we lack the critical datasets to determine whether the step-wise changes that produce the nonlinear patterns correspond with key innovations in phenotype and/or action of selection, and thus we still lack a comprehensive test of this idea. One of the reasons why step-wise events may have not been reported as often as incremental effects is that perhaps they have largely gone undetected, either because we are not looking for them or because the popular model systems for studying major evolutionary transitions do not exhibit the level of social complexity at which the profound shift in the way the genome computes the phenotype occurs. Specifically, most of the popular study organisms exhibit either very simple forms of sociality (stage 1—e.g. *Polistes* paper wasps), or at the other extreme, as truly superorganismal species, with derived traits that have evolved independently of the major transition itself (e.g. *Apis mellifera* honeybees). We are largely lacking genomic data on representatives of the critical evolutionary window around transformation to the new higher-level individual, namely, at the transition from stage 1 to stage 2 (dashed line in figure 2): suitable species include the swarm-founding polistine wasps (e.g. *Metapolybia, Agelaia, Polybia*) [43,79] and the corbiculate and carpenter bees [86]. A critical area for future work will be to fill these gaps.

Another key area of future research lies in understanding *when* and *why* incremental or step-wise processes might be important in the major transition. Future work should focus on interrogating ultimate processes to identify the species and levels of social complexity where we are most likely to detect any step-wise evolutionary events [25,30]. Genomic evolution is likely to arise in response to challenges that require plasticity to factors such as ecological stress [76], environmental stochasticity [87] and changes in resources/nutrition. Genomic change may also correlate with fitness-affecting phenotypic innovations, e.g. loss of mating ability or independent nest founding, evolution of specialist workers and increased colony size in social insects [88]. The molecular machineries underlying these shifts in fitness-affecting phenotypic innovations are key parts of the genome in which to look. Nonlinear changes in molecular mechanisms regulating social behaviour are expected to be confined to species representing the key transitionary 'point of no return', where the evolutionary conditions are right. Indeed, an ultimate framework for understanding *when* we might most expect to see step-wise changes in fitness-affecting traits in social evolution already exists in the form of the monogamy hypothesis, which predicts that an organism will only transition to being superorganismal under a monogamous mating system [25]. Large-scale comparative analyses of genomic traits across spectra of social complexity could be a useful ‘top-down' approach to reveal these key innovations in phenotype and/or units of selection. Of equal importance, however, is a continuing commitment by researchers to understanding natural history as this will help identify species with key phenotypic innovations, and hence prime candidates in which to look for a punctuated genomic event.

In this paper, we have referred to the transition from solitary to superorganismal society in insects. Should we be surprised if multicellularity arises via different macro- and micro-evolutionary patterns and processes to insect superorganismality? Not necessarily: although the different major transitions are united by the same conceptual shift—from lower-level biological entity (e.g. the solitary insect; the single cell; the prokaryote) to a new hierarchical level of higher individuality (e.g. the society; the multicellular organism; the eukaryote)—there are fundamental differences in the drivers of selection. Insects likely require groups of relatives to cooperate and transition to the society (fraternal transitions [89]); other transitions (e.g. from prokaryote to eukaryote) involved unrelated units. The relatedness structure likely affects how the fitness-affecting interactions play out, in the balance of conflict and cooperation in a group. Where the underbelly of selection differs so profoundly, we should not be surprised if evolution invented different ways to derive biological complexity. The quest to discover the evolutionary pathways in major transitions, therefore, may help answer a more basic question: is the concept of the major transition simply a useful classification for us to cluster together a series of evolution's most splendid phenomena under the same conceptual umbrella? Or is the major transition indeed an overarching, common evolutionary process that shapes the complex hierarchies of the biological world?

## 6. Concluding remarks

The ideas posed here have important implications for our empirical understanding of the mechanisms by which sociality and other major transitions may arise. Many theoretical models to explain major transitions assume that selection shapes complex adaptation through the accumulation of many mutations of small effect [7]. If nonlinear patterns of regulating molecular processes are found to be important in permitting the evolution of social complexity in the major transition, new types of theoretical models may be required to understand how biological complexity emerges and is maintained (e.g. [90]). The relative importance of linear and gradual nonlinear processes in major transitions, and the extent to which the same broad-scale patterns and processes can be generalized across the major transitions, require phylogenetically controlled genomic interrogation of a wide range of species, taxonomic groups, levels of complexity and types of major transitions across the tree of life. More broadly, our framework adds to the timely debate over how complexity of life evolves: until recently such studies relied on fragmented fossil records, which supported an incremental model; large-scale genomic data, ecological data and phylogenetically robust statistical models may now reveal evidence of step-wise events [32,91].

## Data Availability

This article has no additional data.

## Glossary

**Major transition in evolution**: A macroevolutionary change in which smaller, formerly independently replicating biological units (e.g. genes, cells, individuals) come together into a larger, co-dependently replicating unit (e.g. chromosomes, multicellular organisms, eusocial societies)

**Superorganism:** A group of individual organisms that form a co-dependent unit, relying on each other for survival and reproduction, that itself can be the target of natural (group-level) selection

**Macro-evolution**: Large-scale evolutionary changes that occur over long periods of geological time and across whole taxonomic groups (e.g. species, families, phyla, etc.)

**Micro-evolution**: Small-scale evolutionary changes that occur over shorter periods of evolutionary time (some finite number of generations) and typically studied within a species or population

**Sociogenomics**: Field of study integrating social behaviour and genomics, concerned with understanding the mechanisms and evolution of social life

**Phenotypic gambit:** The assumption that the evolutionary dynamics of a population can be understood by examining the fitness of phenotypes without consideration of its true underlying genetic basis, or that complex trait evolution can be modelled as if controlled by single alleles

**Nonlinear evolution**: The process describing the phenomenon that evolutionary change occurs suddenly or in rapid bursts, in between periods of gradual change

**Linear evolution**: The process describing slow, relatively steady evolutionary change over long periods of geological time

**Eusocial**: A form of social life involving colony-forming groups containing castes, i.e. designated reproductive individuals as well as sterile, non-reproductive individuals; sometimes also characterized by overlapping generations and cooperative care of the young

**Castes**: Distinct morphological or behavioural forms, specializing on certain functions within a eusocial society

**Polyploidy:** Genomic condition in which additional copies of the entire genome (i.e. entire complement of chromosomes) are carried within each cell of an organism; can vary between cells within the same individual, between individuals and between species

**Genomic insertions**: Occurrence of stretches of DNA nucleotides not previously seen in a genomic region; may originate from within the organism's own genome or from other organisms

**Genomic deletion**: A type of mutation in which stretches of DNA nucleotides previously present in a genomic region are removed from a genome during DNA replication

**Synteny**: Genomic condition in which there are conserved blocks of DNA sequence found across chromosomes of different species or taxa

**Genetic accommodation**: Adaptive change in the environmental regulation or control of a phenotype, which may result in either an increase or a decrease in a phenotype's sensitivity to environmental stimuli over evolution
